# Editorial: Machine Learning in Genome-Wide Association Studies

**DOI:** 10.3389/fgene.2020.593958

**Published:** 2020-10-30

**Authors:** Ting Hu, Christian Darabos, Ryan Urbanowicz

**Affiliations:** ^1^Memorial University of Newfoundland, St. John's, NL, Canada; ^2^Queen's University, Kingston, ON, Canada; ^3^Dartmouth College, Hanover, IN, United States; ^4^University of Pennsylvania, Philadelphia, PA, United States

**Keywords:** GWAS—genome-wide association study, machine learning, complex diseases, gene-gene interaction, epistasis

## Introduction

Genome-wide association studies (GWAS) are used to detect genetic variants that explain common human diseases in populations. The initial GWAS achieved notoriety by successfully identifying thousands of genes associated with a variety of genetic disorders. However, these identified genes have been most successful in establishing individual associations with Mendelian diseases and explaining only a small portion of the heritability. Complex diseases are likely better explained by multiple interacting genetic and environmental variants. Such non-linear, non-additive gene-gene interaction effects, i.e., epistasis, render traditional one-gene-at-a-time analysis methods ineffective for GWAS. Instead, powerful machine learning algorithms that can detect and characterize high-order interactions among multiple genetic variants are needed.

The focus of this Special Topic Issue is to examine the novel design and application of machine learning algorithms in detecting interacting genetic variants for GWAS in six included articles.

Liu et al. proposed a deep-learning framework using convolutional neural networks to predict the quantitative traits from single nucleotide polymorphisms (SNPs) and to investigate genotypic contributions to the trait using saliency maps. The authors evaluated the performance of the proposed approach using both simulation and experimental soybean datasets. The results showed that deep learning modeling can bypass the imputation of missing values and achieve more accurate results for predicting quantitative phenotypes than well-established statistical methods. The authors claim their approach effectively and efficiently identifies significant SNPs and SNP combinations associated with GWAS data.

Zhang et al. presented circLGB, a machine learning-based framework to discriminate circRNA from other lncRNAs. This approach combined commonly used sequence-derived features and three new ones; adenosine to inosine (A-to-I) deamination, A-to-I density, and internal ribosome entry site. circLGB categorizes circRNAs by utilizing a LightGBM classifier with feature selection. In addition, the authors apply circMRT, another ensemble machine learning framework to systematically predict the regulatory information for circRNA, including their interactions with microRNA, RNA binding protein, and transcriptional regulation. Feature sets including sequence-based features, graph features, genome context, and regulatory information features were modeled in circMRT. Experiments on publicly available datasets and lab generated ones showed that the proposed algorithms outperform the available state-of-the-art methods.

In a review article by Nicholls et al., the authors discussed the landscape of ML applications in GWAS by following three components: selected models, input features, and output model performance. The authors focused particularly on the prioritization of complex disease-associated loci and explored the contributions ML has made toward reaching the GWAS end-game with consequent wide-ranging translational impact.

Leem et al. have proposed a permutation method for GWAS, i.e., ENhanced Permutation tests via multiple Pruning (ENPP). ENPP prunes the features in each permutation round if they were determined to be non-significant. Their simulation study showed that the ENPP method could remove about 50% of the features, at the first permutation round, and by the 100th permutation round, 98% of the features were removed. Only 7.4% of the compute time was required, compared to the original unpruned permutation approach. In addition, they applied this approach to a real data set of ~300 K SNPs, to find the association with a non-normal distributed phenotype.

Arabnejad et al. designed a machine learning algorithm, i.e., Nearest-neighbor Projected-Distance Regression (NPDR), in order to detect complex multivariate effects for GWAS. NPDR used a regression formalism that allowed statistical significance testing and efficient control for multiple testing. In addition, the regression formalism provided a mechanism for NPDR to adjust for population structure, which was applied to GWAS data of Systemic Lupus Erythematosus (SLE). The authors also tested NPDR on benchmark simulated genetic variant data with epistatic effects, main effects, imbalanced data for case-control design, and continuous outcomes. NPDR identified potential epistatic and other effects that influence the complex SLE disorder.

Lastly, in the article by Ni et al., ~300 K stomach tissue-specific eSNPs with gastric cancer (GC) risk in three GWAS datasets were investigated. The authors conducted a gene-based analysis to calculate the cumulative effect of eSNPs through a sequence kernel association combined test and Sherlock integrative analysis. At the SNP-level, they identified two novel variants associated with GC risk. Gene-based analyses identified 2 novel susceptibility genes for GC which were significantly overexpressed in GC tissues than in their adjacent tissues and the high expression level of these two genes was associated with an unfavorable prognosis of GC patients. Co-expression genes with these two novel genes in normal stomach tissues were significantly enriched in several cancer-related pathways.

## Author Contributions

All authors listed have made a substantial, direct and intellectual contribution to the work, and approved it for publication.

## Conflict of Interest

The authors declare that the research was conducted in the absence of any commercial or financial relationships that could be construed as a potential conflict of interest.

